# Two-Step Purification of Glycerol as a Value Added by Product From the Biodiesel Production Process

**DOI:** 10.3389/fchem.2019.00774

**Published:** 2019-11-19

**Authors:** Abdul Aziz Abdul Raman, Hooi W. Tan, Archina Buthiyappan

**Affiliations:** Department of Chemical Engineering, University of Malaya, Kuala Lumpur, Malaysia

**Keywords:** biodiesel, crude glycerol, purification, acidification, ion exchange, optimization

## Abstract

For every ton of biodiesel produced, about 100 kg of glycerol is also generated as a by-product. The traditional method of removing glycerol is mainly by gravity separation or centrifugation. This method generates crude glycerol, which may still contain impurities such as methanol, oil, soap, salt, and other organic materials at ppm levels. The effective usage of crude glycerol is important to improve the economic sustainability of the biodiesel industry while reducing the environmental impacts caused by the generated waste. The application and value of crude glycerol can be enhanced if these impurities are removed or minimized. Thus, it is important to develop a method which can increase the economic and applicable value of crude glycerol. Therefore, in the present study, the dual step purification method comprised of acidification and ion exchange techniques has been used to purify the crude glycerol and convert it into higher-value products. The acidification process started with the pH adjustment of the crude glycerol, using phosphoric acid to convert soap into fatty acid and salts. Then, the pretreated glycerol was further purified by ion exchange with a strong cation H^+^ resin. Gas chromatography (GC) was used to analyze both crude and purified glycerol and expressed as the weight percentage of glycerol content. A maximum glycerol purity of 98.2% was obtained after the dual step purification method at the optimized conditions of 60% of solvent, the flow rate of 15 mL/min and 40 g of resin. Further, the glycerol content measured being within the accepted amount of BS 2621:1979. Therefore, this study has proven that the proposed crude glycerol purification process is effective in improving the glycerol purity and could enhance the applicability of glycerol in producing value-added products which bring new revenue to the biodiesel industry.

## Introduction

Biodiesel is a biodegradable and renewable fuel produced by transesterification from renewable sources such as soybean, microalgae, palm cooking oil, and jatropha (da Silva César et al., 2018; Corach et al., 2019). Recently, biodiesel is attracting many researchers as it is one of the most commonly explored biofuel that could reduce the global dependence on fossil fuels and the greenhouse effect. The biodiesel production is estimated to increase annually by 4.5% and reaching 41 Mm^3^ in 2022 (Monteiro et al., 2018).

Crude glycerol is the main byproduct produced during the transesterification process in the biodiesel plant, with the generation of 10 wt.% of the biodiesel product (Samul et al., 2014). Based on the analysis, about 1 kg of crude glycerol is generated with every 10 kg of biodiesel production (Hajek and Skopal, 2010; Tan et al., 2013; Chol et al., 2018). The current market value of pure glycerol is US$ 0.27–0.41 per pound; however, the crude glycerol with 80% purity is as low as US$ 0.04–0.09 per pound. This proved that excessively produced glycerol, affect the price of the glycerol in the market. Therefore, utilization of the crude glycerol for value-added products has become a serious issue in the biodiesel industry.

Glycerol with high purity has a wide application in various industry such as pharmaceutical, cosmetic, and food products. However, the percentage of purity of the glycerol from the biodiesel industry is limiting its conversion to a high valued product (Samul et al., 2014; Talebian-Kiakalaieh et al., 2018). The crude glycerol contains a large number of contaminants such as soap, salts, ethanol, methanol, water, fatty acid, methyl esters, glycerides, and ash (Tan et al., 2013; Dhabhai et al., 2016). Yang et al. (2012) stated that the impurities in the crude glycerol could greatly influence its conversion into other value-added products (Yang et al., 2012). Venkataramanan et al. (2012) also reported that soaps in the crude glycerol have a strong inhibitory effect on the utilization of the glycerol by bacteria, which affects the performance of crude glycerol as the carbon source in the fermentation process (Venkataramanan et al., 2012). As a conclusion, the impurities present in the crude glycerol creates a significant challenge to convert them into a value-added product. Therefore, it is important to purify crude glycerol to avoid market saturation and increase profits of biodiesel production.

In the literature, the most commonly used processes are distillation, ion exchange resin, membrane separation technology, acidification, followed by neutralization and solvent extraction. Acidification is a commonly used technique to neutralize the impurities like catalyst into inorganic salt. Besides acidification, it is also able to reduce the amount of soaps by converting them into insoluble free fatty acid as they can adversely impact the separation and cause loss of yield (Hajek and Skopal, 2010; Kovács et al., 2012). Since acidification process does not remove all impurities, it needs a further purification step to remove other impurities like methanol, oil, water, and ester. However, the distillation process has some limitation over others as it requires high energy input for vaporization and causes thermal decompositions (Lancrenon and Fedders, 2008). Besides, high vacuum is also required in distillation to prevent high-temperature denaturation of glycerol through acrolein formation (Manosak et al., 2011). Furthermore, this process involves high capital investment and maintenance cost, accompanied by considerable losses of glycerol (Sdrula, 2010). In comparison with distillation process, ion exchange process is gaining wide acceptance due to simplicity of operation, low power consumption and energy requirement, as well as the fact it has also proven efficient in removing traces of impurities, color, and odor (Carmona et al., 2009, 2012). Besides, Xiao et al. (2013) suggested that the multiple-step purification process of the crude glycerol could increase the purity make it viable for various usage (Xiao et al., 2013).

This study is aimed to obtain crude glycerol with the highest purity via two-step purification using acidification and ion exchange techniques, with the aid of the Taguchi method. In this study, statistical analysis, including an L_9_ orthogonal array of Taguchi, signal-to-noise ratio, analysis of mean, analysis of variance, and regression analyses were used to identify the optimum conditions of the purification processes.

## Materials, Chemicals, and Methods

### Materials

The crude glycerol was collected from a local biodiesel plant, in Malaysia. A strong cation exchange resin H^+^, Amberlyst 15 was purchased from Sigma Aldrich Sdn. Bhd. The properties of the resin are shown in Table 1. Phosphoric acid (85 wt.%), sodium hydroxide pellets and methanol were purchased from Merck Sdn. Bhd. Distilled water was used for chemical solutions preparation.

**Table 1:** Properties of cation-exchange resins.

**Appearance**	**Dry, spherical beads**
Active group	Sulfonic
Matrix	Styrene—divinylbenzene (macroreticular)
Ionic form	Hydrogen
Particle size (mm)	0.600–0.850
Pore size (nm)	40–80
Surface area (m^2^/g)	50
Bulk density (kg/m^3^)	608
Moisture (by weight)	<1%

*Properties listed in this table were originated in the product specification sheet from resin manufacture*.

### Glycerol Purification Process

#### First Step: Acidification

The crude glycerol was pretreated based on the procedure adopted from Manosak et al. (2011). The experiments were conducted in the 500 ml Erlenmeyer flasks and equilibrated using a magnetic stirrer. Initially, the crude glycerol was acidified by using phosphoric acid to the desired pH value and then stirred at a constant rate of 200 rpm for 1 h. The solution was then left idle for phase separation. It was separated into three layers which are a free fatty acid, glycerol, and inorganic salt layers, respectively. The first layer, which is rich in fatty acid was separated through decantation, and the precipitated salt was removed by filtrations using 0.45 μm filter. The middle layer, which is glycerol-rich, was neutralized (pH 7) by adding NaOH. The inorganic and fatty acid salts that formed in the neutralization stage were removed by 0.45 μm filter. The input parameters selected for this design were pH, temperature, and reaction time, which were designated as parameters A, B, and C, respectively (Table 2). The L_9_ orthogonal array was used to design the experiments in this work (Table 3).

**Table 2:** Operating parameters and levels.

**Parameters**	**Level 1**	**Level 2**	**Level 3**
A:pH	2	4	6
B:Temperature (°C)	30	50	70
C:Reaction time (min)	20	40	60

**Table 3:** L_9_ orthogonal array experimental design and results of the acidification experiments.

**Run**	**pH**	**Temperature (^**°**^C)**	**Reaction time** **(min)**	**Glycerol purity** (wt.%)^*^	**S/N ratio**
	**A**	**B**	**C**		
1	2	30	20	64.12	36.14
2	4	50	40	73.57	37.33
3	6	70	60	76.18	37.64
4	2	50	60	61.16	35.73
5	4	70	20	65.67	36.35
6	6	30	40	67.18	36.54
7	2	70	40	51.37	34.21
8	4	30	60	53.28	34.53
9	6	50	20	58.83	35.39

* *Values are average from the repetition experiments*.

#### Second Step: Ion Exchange

In the ion exchange process, the pretreated glycerol obtained from the acidification process with optimized operating conditions was used. The ion exchange resins were investigated by passing the feed through a 300 ml column of resin-supported in a glass tube. Ion exchange resins type Amberlyst 15 hydrogen form was used for free ions removal. The resin was preliminarily swelled with methanol (25 wt.%) in a glass vessel and packed into the column. Besides, silica beads were also packed inside the column to remove excess moisture content. The ion exchange resins were used to adsorb the free anions and cations in the pretreated glycerol. The pretreated glycerol was then charged into the feed tank, and a pump was used to circulate the crude glycerol through the ion exchange resin bed at the predetermined operating conditions. The temperature of the fixed bed experiment was set up at room temperature (22°C). Then the sample has been put into the rotary evaporator for the methanol removal process. The effluents were collected and analyzed. The input parameters selected for this design were the amount of resin, flow rate and amount of solvents. A standard L_9_ orthogonal array (OA) was selected, and nine experimental studies were performed to optimize the process. The L_9_ orthogonal array is meant for understanding the effect of independent factors, each having 3-factor level values. Taguchi experimental design of experiments suggests L_9_ orthogonal array, where nine experiments are enough to optimize the parameters. Each parameter at three levels for this study is shown in Table 4. Table 5 shows the experimental runs with different combinations of parameters at different levels.

**Table 4:** Operating parameters and levels.

**Parameters**	**Level 1**	**Level 2**	**Level 3**
Amount of resin (g)	30	40	50
Amount of solvent (%)	20	40	60
Flow rate (mL/min)	15	30	45

**Table 5:** L_9_ orthogonal array experimental design and results of the ion exchange experiments.

**Run**	**Amount of resin (g)**	**Amount of solvent (%)**	**Flow rate** **(mL/min)**	**Glycerol purity** (wt.%)^*^	**S/N ratio**
1	20	20	25	89.25	39.01
2	20	40	50	88.13	38.90
3	20	60	75	87.04	38.79
4	30	40	75	90.14	39.10
5	30	60	25	88.68	38.96
6	30	20	50	97.87	39.77
7	40	60	50	88.36	38.93
8	40	20	75	94.89	39.54
9	40	40	25	92.47	39.32

* *Values are average from the repetition experiments*.

### Analytical Methods

Agilent 6890 gas chromatography (GC) attached with a flame ionization detector (FID) was used to identify the concentration of glycerol under the following conditions: (i) capillary column (DB 5HT), 0.32 mm internal diameter, 15 m length with 0.1 μm of liquid film, (ii) carrier gas helium at 1.0 mL/min, and (iii) injector temperature 200°C, and (iv) total run time of 5 min. The water content of glycerol was measured using Karl Fisher titrator. Standard method (ISO 2098-1972) was used to calculate the ash content. The organic non-glycerol (MONG) of glycerol was measured by subtracting the sum of the contents of glycerol, ash and water based on the standard method (ISO 2464-1973). Determinations of pH for the crude and purified glycerol was conducted using a pH meter (Cyberscan pH 300, 19 Eutectic instruments).

### Design of Experiments Using the Taguchi Method

In this study, the Taguchi method was used to design and optimize the crude glycerol two-step purification process. Minitab 16 software package was used to assist the design of experiments and statistical analysis in determining the optimum operating conditions. In this study, glycerol content (wt.%) was used as the parameter to evaluate the effectiveness of the acidification process under different operating conditions. The data obtained for each experiment in OA were analyzed by Signal-to-noise ratio (*S/N* ratios) to investigate the impact of influential factors and determine the optimum configuration of parameters set within the experimental design. The *S/N* ratio can be optimized using several criteria including the larger-the better, the smaller-the better, or the nomina-the better.

In this study, the larger-the better approach was employed to evaluate the experimental response for the purification of glycerol. The S/N ratio was calculated using Equation (1) (Park, 1996; Sharma et al., 2005):

(1) $$\begin{array}{r} \frac{S}{N}=-10\log\left(\;\frac{1}{n}\;\overset{n}{\underset{i=i}{\sum }}\frac{1}{Y_{i}^{2}}\right) \end{array}$$

where “*n*” *represents* total number of replications of each test run and Yi represents the glycerol purity in replication experiment “i” carried out under the same experimental conditions of each test run. The *S/N* ratio was calculated for each experiment. The significant parameters were identified based on the *S/N* ratio of the glycerol purity.

#### Analysis of Mean

In this study, Analysis of Mean (ANOM) was used to determine the optimal operating condition of the acidification process (Chary and Dastidar, 2012). The mean of the S/N ratio shows the effect of each parameter, independently. The mean of the S/N ratio was calculated by averaging the value of the S/N ratio [calculated using Equation (1)] of all the experiments.

The mean of the S/N ratio of an individual parameter “*F*” at level “*I*” was calculated using Equation (2):

(2) $$\begin{array}{r} M_{i}^{F}=\frac{1}{n_{Fi}}\;\overset{n_{Fi}}{\underset{j=1}{\sum }}{\left[{\left(\frac{S}{N}\right)}_{i}^{F}\right]}_{j} \end{array}$$

Where *n*_*Fi*_ is the number of appearences of parameter “*F*” at level “*i*” and ${\left[{\left(S/N\right)}_{i}^{F}\right]}_{j}$ represents the *S/N* ratio of parameter “*F*” at level “*i*” in its j^th^ value (where j = 1,2,3…, *n*).

#### Analysis of Variance

The Analysis of variance (ANOVA) analysis was carried out to statistically assess the effect of different parameters on the performance of the process. ANOVA was performed by calculating the sum of squares (SS), variance (V), degrees of freedom (DOF), variance ratio (F factor), and contribution percentage (ρ_F_). In ANOVA, the significance of all parameters and the interaction among the parameters were investigated using the equations listed below. According to Taguchi method, the percentage contribution of all the studies parameter was used to evaluate the influence of each parameter on the acidification process and to investigate which parameters significantly affected the process response through the ANOVA analysis (Roy, 2001). The percentage contribution of each parameter, ρ_F_, was calculated using the equation below:

(3) $$\begin{array}{r} \rho_{F}=\frac{SS_{F}-\left(DOF_{F}\times V_{e}\right)}{SS_{T}}\times 100 \end{array}$$

In Equation (3), *V*_*e*_ is the variance due to error, *DOF*_*F*_ is the degree of freedom of the studied parameter, and it can be calculated by subtracting one from the number of level of the parameter (*L*).

The sums of squares due to factor, SS_F_ was calculated using Equation (4):

(4) $$\begin{array}{r} SS_{F}=\frac{\sum {\left(\eta_{t}\right)}^{2}}{m}-\frac{{\left(\sum \eta_{i}\right)}^{2}}{n} \end{array}$$

Which, η_*t*_ = the total of the S/N ratio of each parameter in i_th_ level, η_*i*_ is the S/N ratio of the experimental results and *m* is the repeating number of each level of the parameter.

The *SS*_*T*_ in Equation (3) was calculated using Equation (5). SS_T_ is the total of sum squares, *N* is the number of all observations,

(5) $$\begin{array}{r} SS_{T}=\;\sum \eta_{i}^{2}-\frac{{\left(\sum \eta_{i}\right)}^{2}}{n} \end{array}$$

Sum of squares due to error, *SS*_*e*_, was calculated by Equation (6):

(6) $$\begin{array}{r} SS_{e}=SS_{T}-\sum SS_{F} \end{array}$$

The variance of the parameter, *Vp* was calculated by Equation (7):

(7) $$\begin{array}{r} V_{p}=\frac{SS_{F}}{DOF_{F}} \end{array}$$

The Fisher ratio (F) which determines the meaningfulness of a parameter was calculated by Equation (8):

(8) $$\begin{array}{r} F=\frac{V_{p}}{V_{e}} \end{array}$$

### Confirmatory Experiments

Confirmation test was carried out to verify optimal conditions proposed by ANOM and ANOVA analysis. The predicted glycerol purity and S/N ratio were calculated using Equation (9):

(9) $$\begin{array}{r} Y=Y_{m}+\overset{K}{\underset{i=1}{\sum }}\left(\bar{Y_{i}}-Y_{m}\right) \end{array}$$

where *Y*_*m*_ = The total mean of S/N ratio, $\bar{Y_{i}}$ = S/N ratio at the optimal level, and k = number of parameters.

## Results and Discussion

### Characterization of Crude Glycerol

The crude glycerol was a dark brown liquid with a pH of 9.6. It has a higher pH compared to commercial glycerol. The crude glycerol contains a small amount of glycerol (46.8 wt.%), but high ash, water, and MONG content, as can be seen in Table 6. It is shown that the main impurity in the crude glycerol is the MONG content (50.4 wt.%). The MONG is composed of impurities such as soap, alcohol and methyl esters in the glycerol from the biodiesel processing steps (Kongjao et al., 2010). The free fatty acids formed will be released as a soluble soap. Moreover, the methyl esters will be suspended in the glycerol phase during the phase separation process (Kongjao et al., 2010). These organic compounds also possibly react with the excess alkaline catalyst such as NaOH or KOH, which remains in the glycerol solution to reform soap. The ash content (4.7 wt.%) is composed of inorganic matters originating from the utilization of alkali catalysts like NaOH and KOH during the transesterification process. The water content of 9.3 wt.% in the crude glycerol sample is maybe because of the hygroscopic nature of glycerol that absorbs moisture from surrounding during transesterification process.

**Table 6:** Characteristics of crude glycerol obtained from biodiesel production and commercial glycerol.

**Parameters**	**Commercial glycerol^a^**	**Crude glycerol**	**Pretreated** **glycerol^b^**	**Purified glycerol^c^**
Glycerol content (wt.%)	99.98	35.60	77.42	98.20
Ash content (wt.%)	0.002	4.73	0.34	0.39
Water content (wt.%)	0.01	9.38	1.81	0.63
Mong content (wt.%)	0.001	50.29	20.43	0.78
pH	7	9.6	7.05	7.08
Color	Clear	Dark brown	Light brown	Clear

a *Data obtained from the supplier*.

b *Glycerol purity after acidification process*.

c *Glycerol purity after ion exchange process*.

### Acidification

Taguchi method was used to study the effect of parameters on the performance of the acidification process and identify the optimal operating condition. Three controllable parameters (pH, temperature, and reaction time) with each parameter at three different levels were used to design the experiment. Based on the selected parameters, levels and degrees of freedom, a standard L_9_ OA was chosen. Based on the Taguchi method, the results of the experiments were calculated in the term of the S/N ratio and then interpreted. The S/N ratios measure the deviations of the quality characteristics from the desired value and calculate the optimal conditions (Karabas, 2013). The objective of this study is to maximize the glycerol purity. Thus, higher quality characteristics are better desired. Equation (1) was used to determine the S/N ratio. The *S/N* ratios of each experimental run were obtained by substituting the values of glycerol purity and several replicates of each experimental run “*n*” into Equation (1).

#### Optimal Conditions by ANOM Approach

**Figure 1:**
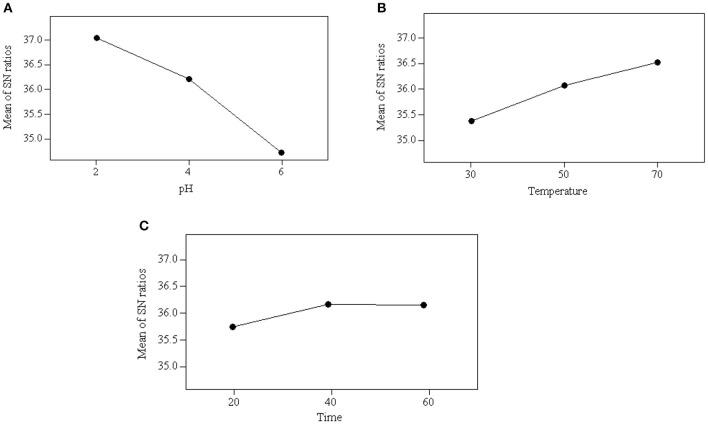
Average value of S/N ratio at level 1–3 of each parameter: **(A)** effect of pH, **(B)** effect of temperature, and **(C)** effect of reaction time.

ANOM is used to identify the effect on the individual parameters and identify the optimum condition for the acidification process (Chary and Dastidar, 2012). This analysis was performed by averaging all the S/N ratios of that particular parameter used in the experiments. Equation (2) was applied to calculate the mean of S/N ratio and the values obtained for each experiment are presented in Table 7. The optimum operating conditions were determined based on the maximum *S/N* ratio at a certain level. The higher mean of S/N ratio indicates that the parameter has a stronger effect on the acidification process. As can be seen Figure 1, the optimum operating conditions for carrying out acidification to obtain the maximum glycerol purity were identified as follows: pH at level 2 (2), reaction temperature at level 3 (70°C) and reaction time at level 2 (40 min). The results obtained from ANOM were further verified by ANOVA.

**Table 7:** Response table of the mean of S/N ratios for glycerol purity.

**Level**	**Mean of S/N ratios**		
	**pH**	**Temperature(^**°**^C)**	**Reaction time (min)**
1	37.04^b^	35.36	35.74
2	36.21	36.07	36.15^b^
3	34.71	36.52^b^	36.07
Delta^a^	2.32	1.16	0.41
Rank	1	2	3

a *Delta represents the deviation of the highest value from the lowest value*.

b *Optimum level of the parameter*.

#### Effect of Parameters on Acidification

The mean of S/N ratios reflects the level of the parameters on the acidification process. As shown in Figure 1, pH is the dominant parameter affecting the acidification process and quality of the product, followed by temperature and reaction time. This indicates that the parameter of pH is critically affected by the acidification process and the quality of a product obtained. The significance of the parameters was also obtained quantitatively from ANOM. It was calculated by calculating the deviation of the highest value from the lowest value. The highest rank was assigned to the parameter that carried the highest value of deviation. A large deviation indicates significant contribution and effect of that particular parameter on the performance of the acidification process. As shown in Table 7, pH was the most significant parameter with a deviation of 2.32 and reaction time was the least significant parameter with a deviation of 0.41.

#### Percentage Contribution of Parameters by ANOVA

According to Taguchi method, the percentage contributed by each parameter was evaluated to accurately quantify the effect of the parameter on acidification in terms of the glycerol purity (Roy, 2001). The results of the ANOVA analysis and the percentage contributions of each parameter is shown in Table 8. It was observed that pH had a dominant effect on the acidification process, with the percentage contribution of 76.37%. The contribution of the parameters was found in the following order: pH (76.37%) > temperature (19.44%) > reaction time (2.72%). This result was in agreement with the results obtained from ANOM analysis.

**Table 8:** Results of ANOVA analysis.

**Parameter**	**Degree of freedom**	**Sum of square**	**Variance**	**F-test**	**Contribution (%)**
pH	2	429.52	214.76	208.95	76.37
Temperature	2	110.85	55.43	53.93	19.44
Reaction time	2	17.27	8.64	8.40	2.72
Error	2	2.06	1.03		1.47
Total	8	559.69			

#### Confirmation Experiments

Confirmation experiment is an important step in the Taguchi design method. This step must be carried out at the end of the optimization study to verify whether the optimized operating conditions, which are identified using ANOM, produce the desired experimental output. The combination of the identified optimal operating conditions was not included in the nine experimental runs of the orthogonal array. As such, a confirmatory experiment was performed for the acidification process by using the optimized value of each parameter and the S/N ratio was calculated. The purity of glycerol was estimated using Equation (9) and the comparison between the actual and predicted glycerol purity is presented in Table 9. As can be seen from Table 9, the S/N ratio obtained from the confirmation experiment is in good agreement with the predicted ones. These results showed that the optimization of the acidification process to yield glycerol of the highest purity was successful.

**Table 9:** Optimal conditions, actual, and predicted value for the response (glycerol purity).

	**pH**	**Reaction temperature (^**°**^C)**	**Reaction time (min)**	**Glycerol purity (wt.%)**		**S/N ratio**	
				**Actual**	**Predicted**	**Actual**	**Predicted**
Optimal condition	2	70	40	77.42	76.58	37.85	37.74

### Ion Exchange

Three controllable parameters (amount of resin, amount of solvent, and flow rate) with each parameter at three different levels were optimized using the Taguchi orthogonal arrays experimental design. Based on the identified number of parameters, several levels, and the degrees of freedom, a standard L_9_ OA was selected in the current study. A total of twenty-seven experimental runs were conducted based on the L_9_ OA with three replications. For each experimental run, the response of the process in terms of glycerol purity (wt.%) was determined and further analyzed by the statistical approach. The collected data on the glycerol purity were presented in Table 5. Based on the obtained results, the glycerol purity (wt.%) of the experiments were found to vary from 87.04 to 97.87 wt.%. This indicates that the ion exchange process is dependent on all controllable parameters (amount of resin, the amount of solvent, and flow rate), and this finding was further proven by the statistical analysis. The results of the experiments were converted into the S/N ratio. This study aims to maximize the glycerol purity obtained from the ion exchange process. Thus, higher quality characteristics [was given in Equation (1)] is used to calculate the S/N ratio.

#### Optimal Conditions by ANOM Approach

**Figure 2:**
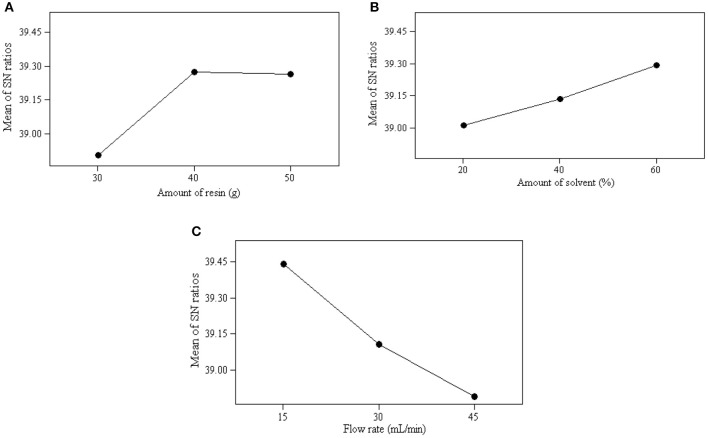
Average value of S/N ratio at level 1–3 of each parameter: **(A)** effect of amount of resin, **(B)** effect of amount of solvent, and **(C)** effect of flow rate.

The mean of S/N ratio obtained for the experiment are presented in Table 10. The optimum operating conditions were selected based on the maximum value of the *S/N* ratio at a certain level of a parameter. A stronger effect on the ion exchange process is indicated by a higher mean of S/N ratio. Therefore, the optimum operating conditions for the parameters were obtained at the level with the largest mean of S/N ratios. As shown in Figure 2, the optimal operating conditions for the ion exchange process to achieve the maximum glycerol purity were identified as follows: the amount of resin at level 3 (40 g), the flow rate at level 1 (15 mL/min), and the amount of solvent at level 3 (60%). The results obtained from ANOM was further verified by ANOVA.

**Table 10:** Response table of the mean of S/N ratios for ion exchange.

**Level**	**Mean of S/N ratios**		
	**Amount of resin (g)**	**Amount of solvent (%)**	**Flow rate (mL/min)**
1	38.56	38.88	39.42^b^
2	39.21	39.04	39.00
3	39.28^b^	39.13^b^	38.63
Delta^a^	0.72	0.26	0.79
Rank	2	3	1

a *Delta represents the deviation of the highest value from the lowest value*.

b *Optimum level of the parameter*.

#### Effect of Parameters on Ion Exchange

The range of the mean of S/N ratios reflects the influence level of the parameters on the ion exchange process. As shown in Figure 2, the flow rate was the dominant parameter affecting the ion exchange process and product quality, followed by the amount of resin and amount of solvent. The significance of these parameters was also be obtained quantitatively from ANOM. It was determined by calculating the deviation of the highest value from the lowest value. The highest rank was assigned to the parameter carrying the highest deviation value. A substantial deviation indicates significant contribution and effect of that particular parameter on the performance of the ion exchange process. As shown in Table 10, the flow rate was the main contributing parameter and the amount of solvent was the least contributing parameter.

#### Percentage Contribution of Parameters by ANOVA

The results of ANOVA on the glycerol purity and the percentage of contributions of each parameter is presented in Table 11. It was clear from the result that flow rate exhibited a dominant effect on the ion exchange process with the percentage contribution of 51.02%. The contribution of the parameters in ascending order as follows: flow rate (51.02%) > amount of resin (28.42%) > amount of solvent (12.33%). The experimental results were in good agreement with the results obtained from the ANOM analysis.

**Table 11:** Results of ANOVA analysis for ion exchange process.

**Parameter**	**Degree of freedom**	**Sum of Square**	**Variance**	**F-test**	**Contribution (%)**
Amount of resin	2	29.61	14.80	14.83	28.42
Amount of solvent	2	13.98	6.99	7.00	12.33
Flow rate	2	51.57	25.79	25.84	51.02
Error	2	2.00	1.00		8.23
Total	8	97.16			

#### Confirmation Experiment

The model predicted 96.91% of glycerol purity, and S/N ratio of 39.72 under the optimal conditions of 60% of solvent, the flow rate of 15 mL/min, and 40 g of resin. The experimentally obtained values were compared with the value predicted by the model to confirm the validity of the optimization procedure under the established operating conditions. The result shows that a maximum glycerol purity (98.2%), and the S/N ratio of 39.78 were obtained using the optimized operating conditions. The results of the confirmation experiments revealed that the actual experimental value and the S/N ratio obtained were in good agreement with the predicted ones. Therefore, it can be concluded that the optimization of the ion exchange process to improve glycerol purity was successful.

### Comparison of the Characteristic of Purified Glycerol With Other Works

The result obtained in this work has been compared with previous studies and presented in the Table 12. Comparison table shows that the dual step purification method comprised of acidification and ion exchange techniques applied in this work successfully produced glycerol with the higher purity compared to other work. The percent of purified crude glycerol obtained from this study was 98%. Saifuddin et al. (2013) achieved lower yield of glycerol with the purity of 93.1–94.2% by using both acidification and adsorption treatment compared to this work. Besides, our two step purification techniques were more effective and superior compared to chemical and physical treatment used by Manosak et al. (2011) and Kongjao et al. (2010) in terms of glycerol purity.

**Table 12:** Comparison of characteristics of purified glycerol with other works.

**Parameters**	**(Hajek and Skopal, 2010)**	**(Manosak et al., 2011)**	**(Kongjao et al., 2010)**	**(Saifuddin et al., 2013)**	**This work**
Glycerol content (wt.%)	86.0	96.20	93.30	94.20	98.20
Ash content (wt.%)	–	2.08	0.05	0.01	0.19
Water content (wt.%)	–	0.06	0.45	10.50	0.63
Mong content (wt.%)	–	1.50	0.56	1.30	0.78
pH	–	–	7.03	7.40	7.08
Color	–	Clear	Light brown	Clear	Clear

## Conclusion

This aim of this work is to obtain high purity glycerol through the two-step purification process with the aid of the Taguchi optimization tool. The acidification process and followed by ion exchange have produced glycerol with the purity of 98.20 wt.%. At the optimized conditions of pH (2), temperature (70°C), and reaction time (40 min), the acidification process has obtained glycerol with a purity of 76.18 wt.%. In the ion exchange process, the pretreated glycerol, which was obtained from the acidification process with optimized operating conditions, was used. The ion exchange process has obtained glycerol with the purity of 98.20 wt.% at the optimized conditions of 60% of solvent, the flow rate of 15 mL/min, and 40 g of resin. The predicted values by Taguchi method was compared with that of the actual experimental results, and the actual result was found to be in good agreement with the predicted result. It is demonstrating that Taguchi was successfully applied to optimize the two-step for purification of biodiesel-derived crude glycerol. This study shows an improvement in the glycerol purity from 35.60 to 98.20 wt.% after optimization of the acidification and ion exchange processes, with the glycerol content being in the amount accepted based on BS 2621:1979.

## Author Contributions

HT: design of the work, experimental work, analysis and interpretation of data, and writing of the manuscript. AA: concept of the study, design of the work, and revising the manuscript. AB: analysis and interpretation of data and writing of the manuscript.

### Conflict of Interest

The authors declare that the research was conducted in the absence of any commercial or financial relationships that could be construed as a potential conflict of interest.
